# Biopolymer-based active packaging with TiO₂ and nano-encapsulated essential oils improves postharvest stability of fresh-cut kale

**DOI:** 10.1016/j.fochx.2025.103141

**Published:** 2025-10-09

**Authors:** Faezeh Mirzajani, Raheleh Ebrahimi, Weria Weisany, Orang Khademi

**Affiliations:** a Department of Agriculture Science and Engineering, SR.C., Islamic Azad University, Tehran, Iran; b Department of Horticulture, Faculty of Agriculture, Shahed University, Tehran, Iran

**Keywords:** Chitosan-based nanocomposites, Titanium dioxide nanoparticles, Essential oil nanocapsules, Biological macromolecules, Active packaging

## Abstract

Fresh-cut kale (*Brassica oleracea*) is highly perishable due to dehydration, enzymatic browning, membrane damage, and microbial spoilage. This study developed an active packaging system using chitosan-based nanocomposite films containing titanium dioxide (TiO₂) nanoparticles and nanoencapsulated essential oils (EO-NCs) from *Thymus daenensis Celak* and *Bunium persicum*. The nanomaterials were synthesized, characterized (SEM, DLS, FTIR, XRD, UV–Vis), and incorporated into absorbent pads for kale storage at 4 °C over 40 days. The packaging significantly reduced weight loss (∼45–55 %), improved chlorophyll retention (∼30–35 %), and preserved membrane integrity (>40 % at day 40). It also maintained phenolics (3.2–3.4 mg GAE/g vs. ∼2.0 in controls), ascorbic acid, and protein (∼25–30 % higher). EO-NCs showed strong antioxidant activity, inhibiting PPO and POD to reduce browning. Mineral retention was enhanced (Mn and Fe ∼20–25 % higher), while microbial growth was suppressed. Overall, TiO₂ and EO-NCs synergistically improved postharvest stability, demonstrating a sustainable strategy for extending shelf life of fresh-cut vegetables.

## Highlights

• Chitosan–EO-NC–TiO₂ nanocomposite films extended kale shelf life by 30–40 days.
• Active films reduced browning, water loss, and microbial spoilage during storage.
• EO-NCs enabled controlled release of antioxidant and antimicrobial compounds.
• TiO₂ nanoparticles enhanced film stability and contributed photocatalytic defense.
• Bioactive packaging preserved chlorophyll, phenolics, and vitamin C in kale leaves.

## 1 Introduction

Kale (*Brassica oleracea* var. sabellica) is a leafy green vegetable renowned for its health advantages and high concentration of vitamins, minerals, antioxidants, and dietary fiber. As a perishable product, fresh-cut kale is highly susceptible to spoilage, making effective packaging critical for preserving its postharvest quality (Acikgoz, 2011). The encapsulation of delicate products, such as kale, in fresh solutions allows their consumption by people over a long period, enhancing quality, safety, and sustainability in the highly dynamic food business (Boerzhijin et al., 2020a). Nanotechnology utilization in nano-packaging is another milestone in the industry, advancing the shelf life of products, food safety, and reducing its impact on the environment (Gupta et al., 2024; Singh et al., 2023a). Kale is a highly nutritious green vegetable that is extremely perishable in storage (Satheesh & Workneh Fanta, 2020a). Traditional packing methods typically fail to preserve the freshness and nutritional integrity of kale (Vargas et al., 2022a). Introducing nano-packaging, which is a cutting-edge technology that comprehensively uses nanoparticles, is a game-changer (Boerzhijin et al., 2020b).

Nano-packaging functions through the incorporation of nanoparticles that have natural antibacterial effects. Silver, zinc oxide, and titanium dioxide are added to packaging materials to inhibit bacteria and fungi, the major agents responsible for the spoilage of fruits (Sandhya., 2010). By inhibiting microbial growth, nano-packaging extends the storage life of kale and maintains it at optimal quality (Wikström et al., 2019). It also facilitates controlled release of the preservative, which improves shelf life and sensory characteristics such as flavor, texture, and aroma, hence increasing consumer acceptance (Wyrwa & Barska, 2017). In addition, nano-packaging reduces its environmental footprint by maximizing material use, minimizing losses, and maintaining barrier integrity, thus supporting zero-waste initiatives and sustainable food supply chains (Kuswandi, 2017; Lopez-Zaplana et al., 2024; Rao Madhushree et al., 2024). It may even make traceability of products and management of supply chains possible, reducing the loss of food and extending freshness to consumers (Guo et al., 2024). This technical and marketing excellence synergy enhances product quality, consumer confidence, and brand loyalty. There are some reports on the essential oil polymeric nanocapsule in this regard. Some of these studies are directed towards the synthesis and application of chitosan-based bioactive formulations containing plant extracts or essential oils for the enhancement of functional properties and food preservation (Evcil et al., 2025; Mouhoub et al., 2024, Mouhoub, Guendouz, El Alaoui-Talibi, Ibnsouda Koraichi and El Modafar, 2025a, Mouhoub, Guendouz, El Alaoui-Talibi, Ibnsouda Koraichi and El Modafar, 2025b).

Titanium dioxide (TiO₂) is ubiquitous because of its photocatalytic and antibacterial activities; upon light exposure, it generates reactive oxygen species that are capable of destabilizing microbial cell membranes and inhibiting spoilage. Similarly, nanocapsules incorporated with added essential oils prolong preservation by prolonged release of their antioxidant and antibacterial components, overcoming volatility and instability issues (Guillard et al., 2018; Vargas et al., 2022b). Overall, nano-packaging is a suitable green technology that welcomes the new preservation issues for foods, ensures food safety, reduces waste, and responds to consumer requests for fresh, safe, and sustainable food (Souza et al., 2024).

Further advancements in nano-packaging could lead to greater efficiency and effectiveness, along with a lower environmental impact (de Sousa et al., 2023). This will allow innovators to discover new avenues for shelf life extension and safety enhancement and encourage sustainable practices in food packaging through interdisciplinary research between different disciplines, including nanoscience and food engineering (Singh et al., 2023b). Nano-packaging is a significant technological breakthrough and intellectual transformation in handling and preserving our food chain (Gutiérrez-Pacheco et al., 2023). This guarantees that fresh, healthy kale and other perishable fruits and vegetables will be consumed safely by the next generation, thus guaranteeing sustainability and resilience (Paśko et al., 2022a). The primary packaging requirements for kale are to improve product safety and quality, extend shelf life and minimize food waste. This paper investigates the alterations in physiological, metabolic, and freshness parameters of fresh-cut kale preserved in intelligent nano-packaging. The package includes nanocapsules containing antibacterial agents or TiO₂ nanoparticles. The future of nano-packaging suggests promising and positive prospects for food preservation (Sagar et al., 2022).

This research advances active food packaging by co-embedding an inorganic photocatalyst (TiO₂ nanoparticles) and sustained-release organic bioactives (essential-oil nanocapsules of *T*. *daenensis* Celak and *B. persicum*) within a single, food-grade chitosan matrix and deploying the hybrid as absorbent pads for fresh-cut kale. The innovation is twofold: first, a synergistic inorganic–organic nano-blend that unifies antimicrobial photocatalysis with controlled antioxidant/anti-browning release and improved O₂/H₂O barrier performance; second, translation into an industry-ready pad format that retrofits easily into commercial packs, overcoming the common gap between lab-scale films and real-world use. Applied to a high-risk leafy green, the dual-nano system targets the principal routes of deterioration—microbial proliferation, oxidative spoilage, and enzymatic browning—and achieves substantially longer shelf life (∼30–40 days vs. ∼10–15 days) while maintaining nutritional and sensory attributes.

The study pursues three linked objectives. First, we synthesize TiO₂ nanoparticles and essential-oil nanocapsules and verify morphology, size distribution, crystallinity, and chemical interactions using SEM, DLS/PDI, FTIR, XRD, and UV–Vis. Second, we fabricate chitosan-based nanocomposite absorbent pads (TiO₂-only, EO-NC-only, and TiO₂ + EO-NCs), quantify encapsulation/loading and short-term release at 4 °C, and validate their functional integrity. Third, we evaluate preservation efficacy during refrigerated storage by quantifying reductions in weight loss and electrolyte leakage, retention of color metrics, chlorophyll *a*/b, and key nutrients (ascorbic acid, phenolics, carbohydrates, protein), together with overall antioxidant capacity, microbial load, and enzyme activities associated with browning and defense (PPO, POD, PAL, SOD, CAT). We further assess mineral stability (P, Ca, Mn, Fe, Cu) and monitor Ti as a tracer for TiO₂ exposure where appropriate, and benchmark single-agent pads against the combined system to determine additive or synergistic benefits on an integrated quality–safety outcome and net shelf-life extension.

## 2 Materials and methods

### 2.1 Plant materials and treatments

Kale was cultivated in a commercial greenhouse located in the Alborz area of Iran. The leaves were swiftly conveyed to the laboratory, where the petioles were excised. The leaves were subsequently cut into 40 × 40 mm portions with a sharp knife, and the freshly cut produce was prepared. *B. persicum* and *T*. *daenensis* Celak were gathered and verified in the Herbarium of Medicinal Plants and Drugs Research Institute, Shahid Beheshti University. The essential oils were extracted with a Clevenger device for five hours and then stored at −20 °C until utilized in nanocapsule production (Salehi et al., 2012).

To conduct this research, kale seeds (Pakan Seed Co.) were grown under controlled conditions to maturity. Homogeneous leaves will be harvested for post-harvest analysis. A factorial experiment will be designed as a completely randomized design (CRD) with 24 treatments, three replications, and five observations per treatment to estimate the effect of nanotechnology-based packaging on fresh-cut kale quality and shelf life. Experimental variables include six types of nanoparticles—carbon quantum dots and nanoparticles loaded with essential oils of *B. persicum* and *T*. *daenensis* Celak for four storage periods (0, 5, 10, and 15 days). Carbon dot nanoparticles will be synthesized, and essential oils will be loaded and encapsulated into nanoparticles that will be added to absorbent pads used in packaging. After harvesting, the kale leaves will be washed, disinfected, dried, and cut into 40 × 40 mm sizes. Ten pieces per treatment will be stored in storage containers with the pads impregnated with nanoparticles and kept at 4 °C. Storage quality attributes will be assessed on specific days, and the data recorded will be subjected to analysis using appropriate statistical software.

### 2.2 Nanostructure synthesis

#### 2.2.1 Essential oil nano capsules

Essential oil nanocapsules (EoNCs) were prepared using a homogenization method with a cylindrical ceramic container (10 cm × 6 cm × 3 mm). A 40 mL chitosan-PVA (both were purchased from Merck Co., Germany) aqueous solution containing 0.5 % acetic acid was transferred to the test container, and the homogenizer head was immersed 1 cm above the container's base. After 10 s of homogenization, 5 mL of an ethyl acetate (Merck Co., Germany) solution containing 30 % essential oil was added, and the process continued for 20 min (Rafati & Mirzajani, 2011). The resultant emulsion was placed in glass containers, sealed with aluminum sheets, and agitated overnight at room temperature at 500 rpm to facilitate the evaporation of the organic solvent. To isolate and precipitate the particles, 10 mL of the sample was subjected to centrifugation at 6000 rpm for 45 min. The supernatant was eliminated, and the pellet was reconstituted with HPLC-grade water. The mixture was subjected to ultrasonication, subsequently followed by multiple centrifugation cycles. The procedure was reiterated thrice before additional analysis.

#### 2.2.2 Titanium dioxide (TiO₂)

To synthesize titanium dioxide nanoparticles (TiNP), 1 g of titanium nitride (Merck Co., Germany) is combined with 50 mL of deionized water at 80 °C. Subsequently, 10 mL of phosphoric acid is incrementally added and allowed to stand for 30 min. The solution was maintained at 50 °C for 150 min. The decolorized solution underwent centrifugation at 6000 rpm for 30 min. The produced nanoparticles were isolated and dehydrated in an oven at 70 °C. The desiccated samples were subjected to a temperature of 400 °C for a duration of 5 h.

### 2.3 Nanostructure characterization

Following nanoparticle synthesis, their physical and chemical properties were analyzed using various techniques. The absorption pattern was examined using a UV-2501PC ultraviolet absorption spectrophotometer (Shimadzu Co., Japan) equipped with tungsten and deuterium lamps, with measurements conducted in a 3 mL quartz cuvette. The morphology and structure of the nanoparticles were studied using a Hitachi SU3500 scanning electron microscope (SEM, Japan) after coating the samples with a 2–5 nm gold layer. Particle size distribution was determined via a Nanophox dynamic light scattering (DLS) system (SympaTEC Co., Pulverhaus, Germany), featuring a 10 mW He—Ne dual-beam laser (632.8 nm wavelength), automatic sample positioning (50–350 μL chamber), and temperature control (5–35 °C), enabling size measurements in the range of 0.1–50,000 nm. The crystalline structure was characterized using a STOE STADI P X-ray powder diffraction (XRD) system (STOE STADI P, Darmstadt, Germany) with a Cu Kα₁ source (λ = 1.5406 Å) and a 2θ scanning range of 1°–80°, with samples placed in aluminum holders. Infrared absorption characteristics of polymer nanoparticles were analyzed using an FTIR-Tensor27 spectrometer (Bruker Co., Ettlingen, Germany) (Mahmoudi et al., 2023; Rafati & Mirzajani, 2011).

The loading capacity of the nanoparticles was assessed utilizing an ultraviolet-visible spectrophotometer. The solution was centrifuged at 24,000 rpm for 45 min. Subsequently, 1 mL of the supernatant was obtained and combined with 5 mL of acetate buffer (pH = 5) (Merck Co., Germany). The absorption was subsequently measured at 285 nm with a spectrophotometer, and the loading capacity was determined. The essential oil content within the nanoparticles was measured following digestion with a hydrochloric acid and alcohol solution. The concentration of essential oil in alcohol was quantified at 295 nm using a spectrophotometer, facilitating the computation of encapsulation efficiency.

#### 2.3.1 Antibacterial study of nanostructure

The antibacterial efficacy of nanoparticles was assessed against *Staphylococcus aureus* ATCC 25923 and *Escherichia coli* ATCC 22312. Aerobic culture was conducted in Mueller-Hinton broth at 37 °C with continuous agitation at 450 rpm. Bacterial growth curves, with and without nanoparticle exposure, were assessed by measuring optical density at 600 nm (OD600). In the absence of defined antibacterial recommendations for nanoparticles, their antistaphylococcal efficacy was evaluated using the microbroth dilution method, adhering to the modified National Committee of Clinical Laboratory Standards (NCCLS, 2005) protocols (Murray et al., 2007). Bacterial inocula were produced from freshly cultivated colonies, standardized to 0.5 McFarland turbidity using normal saline, and subsequently diluted (1:100) in sterile Mueller-Hinton broth before being introduced into the test trays. Nanoparticles were serially diluted in quantities from 0.5 to 256 mg/mL in sterile 96-well microdilution plates using Mueller-Hinton broth. Following incubation at 37 °C for 22 h, the minimum inhibitory concentration (MIC) was determined. The minimal bactericidal concentration (MBC) was ascertained by subculturing 100 μL from wells exhibiting no apparent growth onto nutrient agar plates. The MBC was established as the minimal concentration that eradicated 99.9 % of bacterial strains. All experiments were performed in duplicate (Mirzajani et al., 2013).

The evaluation of the inhibition zone of nanoparticles adhered to the recommendations established by the Clinical and Laboratory Standards Institute (CLSI). A bacterial suspension, calibrated to the 0.5 McFarland turbidity standard, was made in normal saline and evenly distributed on Mueller-Hinton agar with a sterile swab in three orientations. After 10 min, discs containing 10 μg of various nanoparticle samples were positioned on the infected plates. The plates were subsequently incubated at 37 °C for 20 h. Following incubation, the diameter of the inhibitory zone surrounding 0.5 mm discs was measured and documented with a ruler.

### 2.4 Physiological study of traits

#### 2.4.1 Fresh weight variations

All samples and replicates were manufactured with a mass of 100 g and packaged under aseptic conditions. Weight alterations were assessed after a specified duration utilizing a digital scale with an accuracy of two decimal points.

#### 2.4.2 Color measurement

The entire surface of each kale leaf was measured for color in the CIE 1976 Lab color space. RGB images were captured and converted to Lab values. For each bag, five leaves were assessed, and the mean Lab values were recorded as one replicate (Satheesh & Workneh Fanta, 2020b).

#### 2.4.3 Ion leakage and electrolytic content

To evaluate electrolyte leakage, 0.2 g of fresh kale from each replicate was meticulously washed and placed in glass containers with lids holding 10 mL of ionized water. The containers were maintained at room temperature for 26 h, following which the electrical conductivity (EC) was assessed using an HI2315 desktop EC meter (Hanna Instruments Ltd., Italy) with a measuring range of 0.0 to 199.9 mS (Kale et al., 2007).

### 2.5 Elemental and metabolite analysis

#### 2.5.1 Mineral and elemental analysis

The elemental composition, comprising potassium, phosphorus, calcium, iron, copper, manganese, and titanium, was ascertained by the acid digestion method utilizing strong hydrochloric acid and nitric acid (Merck Co., Germany) at 300 °C. The elemental composition of plant tissue was assessed by Inductively Coupled Plasma Mass Spectrometry (ICP-MS, ELAN 6100 DRC-e, Perkin Elmer Co.). This technique enables the quantification of over 70 elements in the parts per billion (ppb) range, employing Dynamic Reaction Cell (DRC) technology to reduce polyatomic interferences (Acikgoz, 2011).

#### 2.5.2 Pigment concentration analysis

To ascertain total chlorophyll concentration, 20 mg of a dried sample (dehydrated for 3 days at 60 °C) was combined with 2 mL of DMSO (Merck Co., Germany) and subjected to heating at 70 °C for 60 min. After chilling, the sample underwent centrifugation at 8000 rpm for 5 min, and the absorbance of the supernatant was quantified at 470, 647, and 663 nm. The concentrations of chlorophyll *a* (Cla), chlorophyll *b* (Clb), total chlorophyll (ClTotal), and total carotenoids were derived from prior studies. (Mirzajani et al., 2013).

#### 2.5.3 Ascorbic acid concentration analysis

Ascorbic acid was analyzed using a Kiazist Co. assay kit (Iran). In brief, 300 mg of plant tissue was pulverized in 500 μL of lysis buffer and centrifuged (15,000 ×*g*, 10 min). The supernatant was mixed with 20 μL of sulfuric acid (100 μL), and then combined with oxidizing and developing reagents (12 μL). The compound incubated, for 5 min and the absorbance was measured at 500–540 nm (Acikgoz, 2011).

#### 2.5.4 Total polyphenol concentration analysis

Total phenolic content was measured using gallic acid (Merck Co., Germany) as the standard. 100 μL of plant extract (2 mg/mL) was combined with 500 μL of 10 % (*v*/v) Folin-Ciocalteu reagent. After 5 min, 500 μL of 7 % sodium carbonate was added to the mixture. The samples were incubated in the dark for 2 h, and the absorbance was measured at 765 nm (Paśko et al., 2022b).

#### 2.5.5 Antioxidant capacity measurement

The DPPH radical scavenging ability of each extract was assessed using a modified Brand-Williams technique. DPPH radicals, demonstrating peak absorption at 515 nm, diminish their absorbance when reduced by an antioxidant agent. A DPPH• solution in methanol (6 × 10^−5^ M) was made fresh each day. 200 μL of this solution was combined with 100 μL of plant extract solution in a 96-well plate. The samples were incubated at 25 °C for 20 min with stirring at 450 rpm, and the decrease in absorbance at 515 nm was measured. All experiments were conducted in duplicate (Paśko et al., 2022b).

#### 2.5.6 Total carbohydrate concentration analysis

The phenol‑sulfuric acid colorimetric method, recognized as one of the most straightforward and dependable procedures for quantifying reduced sugar content in a liquid media, was employed. Glucose functioned as the reference standard. 50 μL of the sample solution was dispensed into each well, followed by the addition of 150 μL of pure sulfuric acid (98 %) (Merck Co., Germany). Subsequently, 30 μL of a 5 % aqueous phenol solution was introduced. The mixture was subsequently incubated in a water bath at 90 ± 1 °C for 5 min, followed by cooling to room temperature in a separate water bath for 5 min. The color intensity, reflecting carbohydrate concentration, was assessed with a Powerwave spectrophotometer at 490 nm. A 50 μL glucose standard (Merck Co., Germany) solution was employed to construct the standard curve (Mirzajani et al., 2013).

### 2.6 Enzymatic variations analysis

#### 2.6.1 Catalase enzyme activity

The activity of the catalase (CAT) enzyme was assessed utilizing an assay kit from Kiazist Co., Iran. Twenty microliters of the sample, standard, and control were combined with one hundred microliters of X1 Buffer Assay Catalase and thirty microliters of Methanol Catalase, followed by comprehensive mixing. Subsequently, 20 μL of substrate catalase was introduced, and the reaction mixture was incubated at 25 °C for 20 min. Following incubation, 30 μL of Stop Solution Catalase and 30 μL of Chromogen Catalase were introduced to each well. The wells were incubated at 25 °C in darkness for 10 min. Subsequently, 10 μL of periodate catalase was introduced, and after 5 min, the absorbance was recorded at 520–560 nm (Acikgoz, 2011).

#### 2.6.2 Superoxide dismutase enzyme activity

The activity of the superoxide dismutase (SOD) enzyme was assessed utilizing an analytical kit from Kiazist Co., Iran. This kit concurrently identifies both cytoplasmic and mitochondrial forms of SOD in eukaryotic cells. 10–20 mg of fresh plant tissue was homogenized with a probe homogenizer in PBS buffer supplemented with a protease inhibitor cocktail. The homogenate was subsequently centrifuged at 12,000 rpm for 15 min at 4 °C, and the resultant supernatant was preserved at −80 °C until analysis. For the assay, 20 μL of the sample (from a total volume of 100 μL) was combined with SOD Assay Reagent and incubated at 37 °C for 20 min prior to measurement (Acikgoz, 2011).

#### 2.6.3 Phenylalanine ammonia lyase (PAL) enzyme activity

To assess phenylalanine ammonia lyase (PAL) enzyme activity, 0.5 mL of 10 mM phenylalanine solution, 0.4 mL of double-distilled water, 0.1 mL of extracted protein, and 1 mL of 50 mM Tris-HCl buffer (pH 8.8) were added to a test tube. A control sample was produced by incorporating all reagents except the protein extract. The reaction mixture was incubated at 37 °C for 1 h, the ideal temperature for PAL activity. To terminate the reaction, 0.5 mL of HCl was added to inactivate the PAL enzyme. The sample was subsequently rinsed with 15 mL of ethyl acetate and then subjected to evaporation under airflow. The residue was solubilized in 3 mL of 0.05 M sodium carbonate (Na₂CO₃) until completely dissolved. The absorbance of the sample, related to enzyme content (mg/mL), was assessed at 550 nm (Acikgoz, 2011).

#### 2.6.4 Peroxidase (POD) enzyme activity

The activity of the peroxidase enzyme was assessed by combining 50 μL of enzyme extract with 3 mL of measurement buffer, 4.51 μL of H₂O₂, and 3.35 μL of guaiacol. The absorbance variation at 470 nm was documented during a duration of 2 min. Peroxidase activity was quantified by the generation of tetraguaiacol from guaiacol in the presence of hydrogen peroxide and the peroxidase enzyme. In this reaction, peroxidase facilitates the decomposition of hydrogen peroxide utilizing guaiacol as an electron donor, resulting in the creation of tetraguaiacol. The specific activity of peroxidase was quantified as micromoles of tetraguaiacol produced per minute per milligram of protein (Acikgoz, 2011).

#### 2.6.5 Polyphenol oxidase (PPO) enzyme activity

PPO activity was assessed using a reaction mixture comprising 1 mL of 0.05 M phosphate buffer (pH 7.0), 3.3 μL of 30 % hydrogen peroxide, and 1 μL of guaiacol solution. The control sample included 1 mL of 0.05 M phosphate buffer, 1 μL of 30 % hydrogen peroxide, 1 μL of guaiacol solution, and 16.6 μL of enzyme extract. Enzyme activity was quantified by monitoring absorbance variations at 270 nm over a duration of 10 min and expressed as activity per mg of protein (Acikgoz, 2011).

### 2.7 Statistical analysis

Upon completion of the studies, a comprehensive analysis of variance (ANOVA) was performed on the data amassed over two years utilizing SAS statistical software, version 9.1 (SAS Institute Inc., 2009). Before doing ANOVA, the normality of the data distribution was evaluated with the Shapiro-Wilk test to confirm the appropriateness of parametric statistical methods. Mean comparisons were conducted with Duncan's multiple range test (DMRT) at a 5 % significance threshold. Furthermore, figures were produced utilizing Microsoft Excel.

## 3 Results

Kale is a leafy, perishable crop whose post-harvest handling activities greatly influence its cosmetic quality, nutritional quality, and marketability. Traditional packaging activities are unable to reliably ensure the biochemical and physical properties' sustainable maintenance, thereby leading to nutrient loss, contamination by microorganisms, and weight loss during storage. To tackle these problems, unique active packaging techniques utilizing nanoparticles and nanocapsules were investigated as preservation options. The research evaluated essential post-harvest quality metrics, encompassing weight loss, color stability, nutritional preservation, enzyme activity, microbial load, and antioxidant capacity. The utilization of nanoparticles and nanocapsules exhibited promise in prolonging kale's shelf life, maintaining its biochemical integrity, and mitigating degradation effects relative to traditional storage techniques.

### 3.1 Synthesis and analysis of nanostructures

**Fig. 1:**
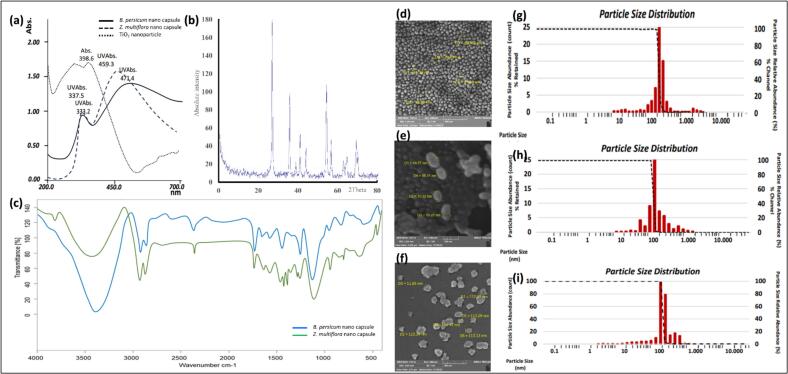
**(a)**: The UV–visible analysis of polymeric nano capsule. **(b)**: The X-ray powder diffraction of TiO_2_ nanoparticle. **(c)**: The Fourier transform infrared (FT-IR) analysis of the chitosan-PVA nano capsule formation. The scanning electron microscope (SEM) results of **(d)**: *B. persicum* essential oil loaded nano capsule, **(e)**: *T*. *daenensis* Celak essential oil loaded nano capsule and **(f)**: TiO_2_ nanoparticle. The dynamic light scattering (DLS) analysis of **(g)**: *B. persicum* essential oil loaded nano capsule, **(h)**: *T*. *daenensis* Celak essential oil loaded nano capsule and **(i)**: TiO_2_ nanoparticle.

The synthesized nanoparticles were comprehensively characterized by a range of analytical techniques for verification of their formation, structure, and morphology. The nanocapsules of polymers were characterized by UV–visible spectroscopy, where the absorbance spectra showed characteristic peaks indicating successful encapsulation of the essential oils within the chitosan–PVA matrix. The intense band of absorbance in the UV region can be regarded as a measure of the homogeneity and stability of the nanoemulsion, possibly due to some interaction between the entrapped molecules and the polymeric coating (Fig. 1A). Some peak shifting of the absorptions, if present, may also be a measure of some bonding or encapsulation in the nanoformulation. The x-ray powder diffraction (XRD) pattern, (Fig. 1B) of TiO₂ nanoparticles showed sharp and well-defined peaks, indicative of high crystallinity. All peak positions match the anatase phase of TiO₂, as indicated by the comparison of the 2θ values with standard JCPDS reference data. This crystalline phase is useful for applications since it is known to be photocatalytic and antibacterial. The narrow peak widths also suggest small crystallite sizes, which can be estimated quantitatively using the Scherrer equation to determine the nanoscale particle sizes.

Fourier-transform infrared spectroscopy (FT-IR) was conducted in order to evaluate the chemical interaction in the polymeric matrix of the chitosan–PVA nanocapsules. FT-IR spectra showed the typical bands of chitosan and PVA with a broad O—H and N—H stretching band around 3400 cm^−1^, C—H stretching peaks around 2920–2850 cm^−1^, and a strong amide I band around 1650 cm^−1^, indicating the existence of chitosan. Other peaks that are characteristic of PVA, such as those in the range 1080–1150 cm^−1^ (C–O–C and C—O stretching), were a sign of successful blending (Fig. 1C). The presence of shifts or changes of these peaks is a sign of polymer–polymer and polymer–essential oil interaction, which confirms successful preparation of the nanocapsules. Scanning Electron Microscopy (SEM) was used to observe the morphology and surface characteristics of the nanocapsules and the TiO₂ nanoparticles. SEM micrographs of *B. persicum* (Fig. 1d) and *T*. *daenensis* Celak (Fig. 1e) essential oil-loaded nanocapsules presented, in general, spherical shapes with smooth surfaces, which were indicative of well-structured and stable encapsulation. The capsules were comparatively uniform in shape, although some degree of agglomeration was noticed, which is relatively common in polymeric systems. In contrast, the TiO₂ nanoparticles (Fig. 1f) presented smaller granular structures, according to the morphology of this type of inorganic nanoparticle. The relatively uniform size and shape of the particles indicate a controlled synthesis process.

Dynamic Light Scattering (DLS) (Fig. 1g, h & 1i) provides a measure of the hydrodynamic diameter and distribution of nanocapsules and TiO₂ nanoparticles when in suspension. *B. persicum* and *T*. *daenensis* Celak essential oil-loaded nanocapsules had size distributions typically in the low nanometer range, with *Z*-average sizes reflecting their swollen, hydrated state in solution. Polydispersity Index (PDI) values obtained from DLS provided further data on the uniformity of the particles. Values below 0.3 are indicative of a narrow size distribution and good formulation stability, whereas higher values suggest broader distribution and potential aggregation. The TiO₂ nanoparticles also had relatively small hydrodynamic sizes with acceptable PDI values, although their DLS sizes were slightly bigger than their SEM-measured sizes due to the solvation effect.

### 3.2 Physiological study of kale: variations in weight, colors and electrolyte leakage

#### 3.2.1 Weight loss and moisture retention

At day 20, EO-NC samples (Fig. 3A) weighed 35 % less than the control, while at day 40, EO-NC still showed considerably lower loss (around 60 % of control values). Weight loss was reduced significantly by EO-NC and TiO₂ nanoparticle treatment, with the greatest reduction observed in the EO-NC group. This obstacle would have reduced water vapor loss and maintained tissue hydration. TiO₂ nanoparticles also facilitated water retention, possibly by creating a thin film on the leaf surface. Generally, treatments preserved moisture and shelf life of kale more than in the control and free essential oil treatments.

#### 3.2.2 Color stability and chlorophyll retention

**Fig. 2:**
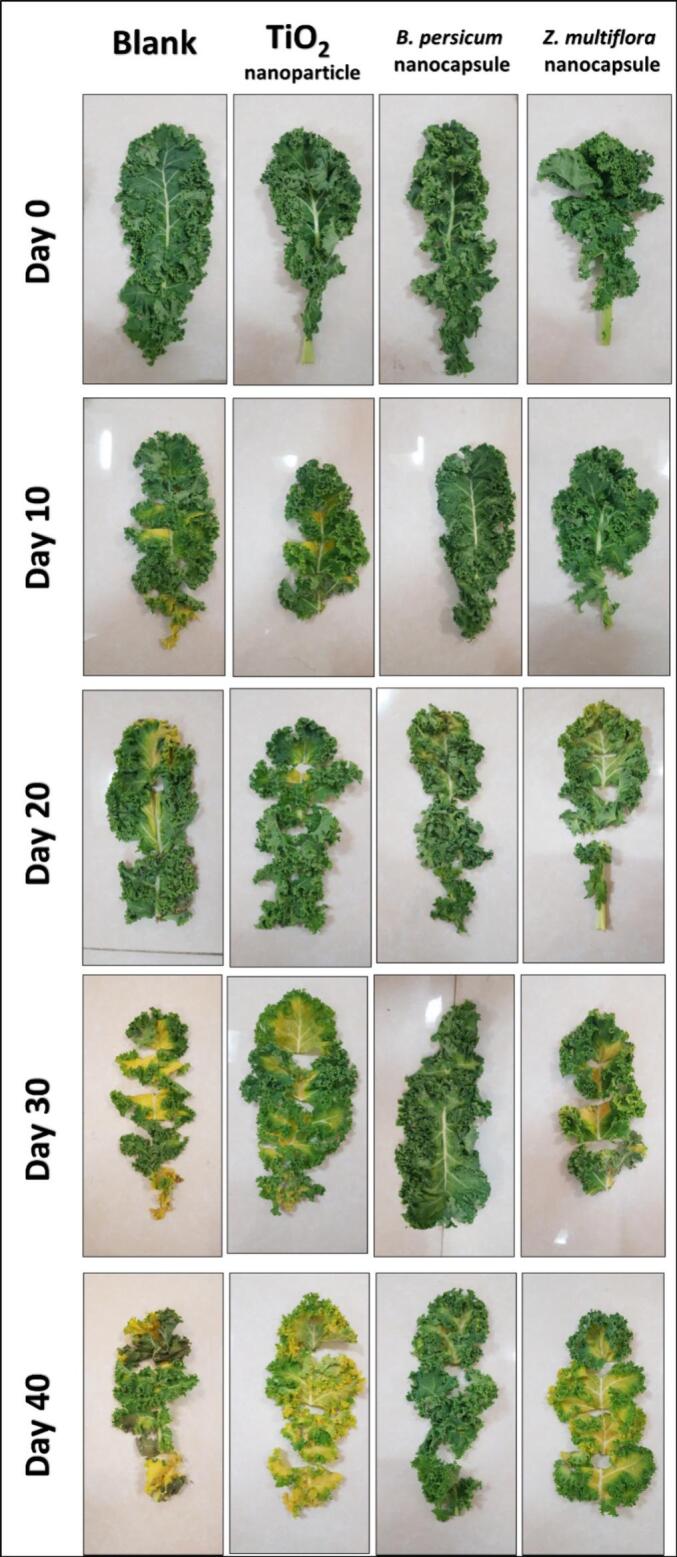
Color changes of kale leaf samples in control conditions compared to three samples stacked with bread and ingredients over 40 days at 10-day intervals**.**

**Fig. 3:**
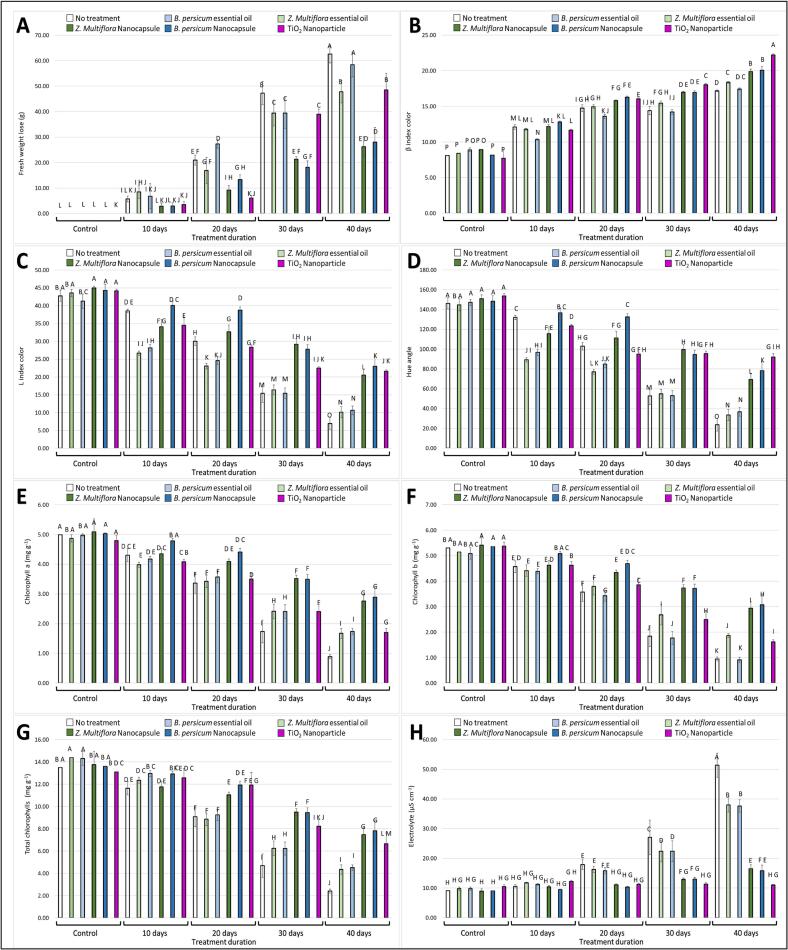
Effect of various treatments on the preservation of fresh-cut kale over different storage durations (0, 10, 20, 30, and 40 days). The figure shows the results for different parameters: **(A)** Fresh weight loss, **(B)** Δβ (yellow color), **(C)** Lightness (L*), **(D)** Hue angle, **(E)** Chlorophyll a content, **(F)** Chlorophyll b content, **(G)** Total chlorophyll content, **(H)** Electrolyte leakage. Treatments include no treatment (control), essential oils (*T*. *daenensis* Celak and *B. persicum*), nanoencapsulated essential oils (EO-NC), and TiO_2_ nanoparticle. Different letters represent significant differences between treatments. (For interpretation of the references to color in this figure legend, the reader is referred to the web version of this article.)

Chlorophyll retention and color attributes are strong indicators of appearance and nutritional quality during storage in leafy vegetables like kale (Fig. 2). Color stability differed significantly between treatments in terms of yellowing (Δβ) change (Fig. 3B), lightness (L*) (Fig. 3C), and hue angle (Fig. 3D), during storage.

At day 20, EO-NC-treated samples, particularly those containing *T*. *daenensis* Celak and *B. persicum*, maintained significantly higher L* values compared with the untreated control, indicating better lightness retention. By day 40, all treatments exhibited a reduction in L* values due to progressive darkening; however, the decline was most pronounced in the control group. In contrast, EO-NC and TiO₂ nanoparticle treatments retained higher L* values throughout storage, representing slower discoloration and improved maintenance of fresh appearance. Regarding Δβ values, untreated control samples showed a sharp increase by day 40, reaching nearly twice the values observed in EO-NC-treated leaves. Both EO-NC and TiO₂ nanoparticle treatments displayed a more gradual increase in Δβ, confirming enhanced retention of green coloration compared to the control. The color change, as expressed by the green-to-yellow color angle, decreased linearly in untreated samples once again confirming visual degradation. However, TiO₂ nanoparticle addition and EO-NC treatments slowed down this change considerably, as evidenced by improved preservation of the green color. This activity was supported by chlorophyll content measurement: chlorophyll *a* (Fig. 3E) and chlorophyll *b* (Fig. 3F) levels were higher in treated tissues compared to control, with maximum retention being achieved with EO-NC treatments. Total chlorophyll content (Fig. 3G) showed the same trend, where nanoencapsulated essential oils were superior to non-encapsulated oils, which suggests that encapsulation improved stability and controlled release of active constituents that may be antioxidant and protective. In brief, these findings point to the fact that EO-NC and TiO₂ nanoparticle treatments effectively delayed chlorophyll degradation and browning and, consequently, increased the extended freshness and marketability of kale upon storage.

#### 3.2.3 Electrolyte leakage and membrane integrity

Electrolyte leakage is a widely used indicator of membrane integrity and reflects the physiological condition of plant tissue. In untreated kale, electrolyte leakage increased sharply during the 40-day storage period, indicating progressive membrane breakdown and cell lysis (Fig. 3H). This corresponded with reductions in firmness, water content, and overall freshness. In contrast, both EO-NC and TiO₂ nanoparticle treatments significantly reduced electrolyte leakage compared with the control. Among them, EO-NC treatments—particularly those containing *T*. *daenensis* Celak —recorded the lowest electrolyte leakage values throughout storage, demonstrating superior preservation of membrane stability. TiO₂ nanoparticle treatment also effectively minimized electrolyte leakage, likely due to its antimicrobial activity, which suppresses microbial spoilage and delays senescence-associated physiological changes. The statistically significant differences among treatments confirm the capacity of EO-NCs and TiO₂ nanoparticles to maintain membrane function more effectively than free essential oils or untreated samples.

The strength of EO-NCs lies in their ability to provide controlled and sustained release of bioactive compounds, which enhances their stability, bioavailability, and functional performance compared with free essential oils. In this study, EO-NCs—particularly those containing *T*. *daenensis* Celak —demonstrated superior efficacy in maintaining membrane integrity, as evidenced by the lowest electrolyte leakage values throughout storage. This controlled release mechanism not only preserved antioxidant activity over time but also offered prolonged protection against oxidative stress and enzymatic browning, thereby delaying tissue senescence. Moreover, EO-NCs were more effective than free essential oils in retaining chlorophyll pigments and maintaining overall freshness, underscoring their potential as a powerful and natural strategy for extending the shelf life of fresh-cut produce.

#### 3.2.4 Statistical analysis and treatment efficacy

The ANOVA statistical analysis revealed that most of the parameters measured were influenced significantly by the treatments administered, as indicated by the highly significant (*p* < 0.01) values in the Model and CKTR (treatment) sources (Table 1). For fresh weight loss, *color indices (α, β, L, hue angle)**, chlorophyll content, electrolyte leakage, and most significant biochemical properties such as ascorbic acid, phenolic content, and total carbohydrates, the treatment model showed high statistical significance, which was a well-established effect of the treatments used on kale preservation. Phenolic content had significantly higher phenolic content than the control (p < 0.01). Interestingly, EO-NC and TiO₂ nanoparticles performed better than control and free EO treatments consistently for all the biochemical and quality parameters. Coefficient of variation (CV%) values were mostly low (<10 % for most of the properties), which means experiments were of high precision.

**Table 1:** ANOVA interaction analysis for TiO2.

Source	DF	Fresh weight loss (g)	α index color	β index color	L index color	Hue angle	Ascorbic acid content (mg)	Electrolyte (μS cm^−1^)	Total Phenol (μg ml^−1^Galic acid)	Total Carbohydrate (g/100 ml Glucose)
Rep	2	8.55 ns	353,783.22 ns	0.44 ns	9.42*	81.88*	4.56 ns	6.48 ns	120.69 ns	0.02 ns
Model	31	1067.85**	8,509,777.3**	43.88**	351.02**	4035.73**	956.89**	287.22**	17,373.65**	4.75**
Block	2	8.55 ns	353,783.22 ns	0.44 ns	9.42*	81.88*	4.56 ns	6.48 ns	120.69 ns	0.02 ns
CKTR	20	156.10**	516,804.58 ns	2.80**	38.42**	609.38**	51.05**	135.90**	6345.76**	1.06**
Error	58	10.61	452,680.4	0.1	1.91	22.13	10.16	3.22	44.66	0.006
CV (%)	–	16.28	2.67	2.21	4.82	4.75	8.08	11.09	3.26	3.39
Source	DF	Total Protein (BSA) mg ml^−1^	Catalase Activity (U mg^−1^ FW)	Phenylalanine ammonia lyase (U mg^−1^ FW)	Polyphenol oxidase (U g^−1^ FW)	Polyphenol peroxidase (U g^−1^ FW)	Superoxide dismutase (U mg^−1^ FW)	Microbial content (colony count)	Antioxidant activity (IC50 against BHT) %	Total chlorophylls (mg/g)
Rep	2	0.08 ns	0.005 ns	0.005 ns	0.003 ns	0.005 ns	5032.68 ns	6134.10 ns	3.60 ns	0.51 ns
Model	31	10.26**	0.57**	0.144**	0.68**	0.37**	400,833.03**	1,321,357.80**	519.12**	32.48**
Block	2	0.08 ns	0.005 ns	0.005 ns	0.003 ns	0.005 ns	5032.68 ns	6134.10 ns	3.60 ns	0.51 ns
CKTR	20	2.23**	0.09**	0.02448**	0.08332**	0.03536 ns	85,011.29**	597,274.95**	220.15**	3.85**
Error	58	0.05	0.00228	0.0031	0.00135	0.02476	2168.8	7140.82	2.66	0.36
CV (%)	–	4.33	3.82	7.16	3.59	18.33	3.37	15.24	3.81	6.12
Source	DF	Chlorophyll a (mg/g)	Chlorophyll b (mg/g)	K (ppm)	P (ppm)	Fe (ppm)	Ca (ppm)	Cu (ppm)	Mn (ppm)	Ti (ppm)
Rep	2	4.08**	5.39**	468,510.41 ns	19,048.01**	55,951.76**	353,783.22 ns	2788.19 ns	4.95 ns	0.0018 ns
Model	31	0.11 ns	0.16*	2,911,413.90**	63,657.34**	20,195,155.10**	8,509,777.3**	51,417.19**	3884.43**	1.13**
Block	2	0.32**	0.49**	468,510.41 ns	19,048.01**	55,951.76**	353,783.22 ns	2788.19 ns	4.95 ns	0.0018 ns
CKTR	20	0.04	0.05	1,351,077.20 ns	12,685.88**	796,149.31**	516,804.58	2127.50**	268.13**	0.6777**
Error	58	5.53	6.1	1,047,038.8	3040.22	10,123.7	452,680.4	900.84	10.69	0.00945
CV (%)	–	4.08**	5.39**	2.68	1.17	1.32	2.666385	14.46	4.22	11.83

Activities of enzymes such as catalase, phenylalanine ammonia lyase, polyphenol oxidase, and superoxide dismutase were also radically different among treatments, verifying the physiological impact of the treatments. Besides, microbial load, antioxidant capacity, and mineral content (P, Fe, Ca, Mn and Ti) were also impacted significantly, EO-NC and TiO₂ once more demonstrating the optimal performance in maintaining postharvest quality and averting microbial contamination.

### 3.3 Mineral content in fresh-cut kale

#### 3.3.1 Phosphorus (P)

**Fig. 4:**
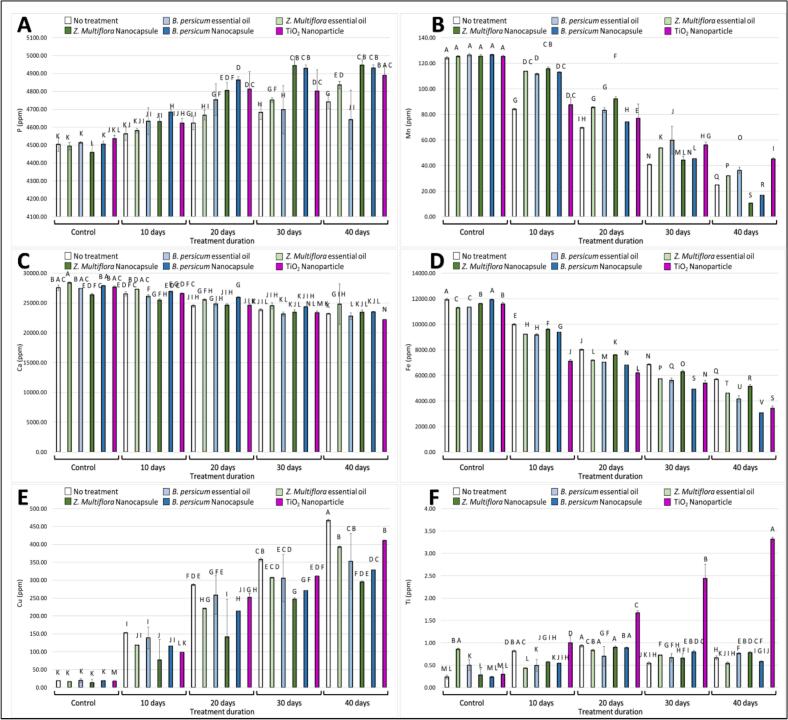
Effect of various treatments on the preservation of fresh-cut kale over different storage durations (0, 10, 20, 30, and 40 days). The figure shows the results for different parameters: **(A)** phosphorus (P) content, **(B)** magnesium (Mn) content, **(C)** calcium (Ca) content, **(D)** Iron (Fe) content, **(E)** cupper (Cu) content, **(F)** titanium (Ti) content. Treatments include no treatment (control), essential oils (*T*. *daenensis* Celak and *B. persicum*), nanoencapsulated essential oils (EO-NC), and TiO_2_ nanoparticle. Different letters represent significant differences between treatments.

Phosphorus (P) content is presented in Fig. 4A. During the storage period, P content decreased in all groups; however, the degree of decrease differed between treatments. The EO-NC and TiO₂ nanoparticle treatments were most effective in sustaining phosphorus content in fresh-cut kale, with significantly higher levels than the control and essential oil-only treatments. These results highlight the importance of advanced preservation methods in reducing nutrient loss during long-term storage.

#### 3.3.2 Manganese (Mn)

Manganese (Mn) content of fresh-cut kale is shown in Fig. 4B, though labeled incorrectly as “magnesium (Mn)” in the figure legend. By day 30, Mn retention in EO-NC and TiO₂-treated kale was nearly 25 % higher than in controls, and this advantage continued through day 40. However, the EO-NC-treated and TiO₂ nanoparticle-treated samples showed considerably greater retention of Mn content compared to the untreated control and essential oil-alone treatment (*B. persicum* and *T*. *daenensis* Celak). This indicates that EO-NC and TiO₂ play significant roles in inhibiting the oxidative or enzymatic degradation of Mn during storage.

#### 3.3.3 Calcium (Ca)

Calcium content variation is presented in Fig. 4C. Ca content gradually decreased over the course of 40 days' storage in all the samples. TiO₂ nanoparticle-treated and EO-NC-treated kale, however, had a much higher content of Ca compared to the essential oil treatment group and the control. These findings suggest that the treatments mentioned above may well inhibit cellular degradation and maintain tissue structure in plant tissue, potentially leading to higher Ca retention.

#### 3.3.4 Iron (Fe)

Fe content in fresh-cut kale is shown in Fig. 4D. There was a decrease in Fe content during the storage period in all the treatments, but the decline was higher in the control samples. *T*. *daenensis* Celak EO, EO-NC, and TiO₂ nanoparticles treatments had Fe content retained considerably, particularly EO-NC, which possessed the highest retention at every moment. This enhanced preservation could be caused by the protective effect of these treatments against enzymatic oxidation or microbial activity.

#### 3.3.5 Copper (Cu)

Fig. 4E indicates the trend in Cu content (labeled as “cupper (Cu)” in the figure). Similar to other minerals, Cu levels decreased over the period, the most significant decrease being in the untreated control samples. EO-NC treatment and TiO₂ nanoparticles lowered Cu loss substantially, maintaining significantly higher levels for the entire 40-day storage duration. The treatments are likely to have provided antioxidative protection or inhibited cell wall breakdown, hence maintaining the mineral content.

#### 3.3.6 Titanium (Ti)

Titanium content is indicated in Fig. 4F. As expected, Ti was found only in TiO₂ nanoparticle-treated samples since it is not a naturally occurring mineral in kale. The Ti content remained stable over time in such samples, confirming the stability of TiO₂ on the plant surface throughout the storage period. This highlights its potential role not only as a preservative treatment but also stresses the importance of monitoring potential accumulation and safety parameters of nanoparticle-based treatments in food systems.

### 3.4 Metabolite and microbial activity

#### 3.4.1 Total phenolic content

**Fig. 5:**
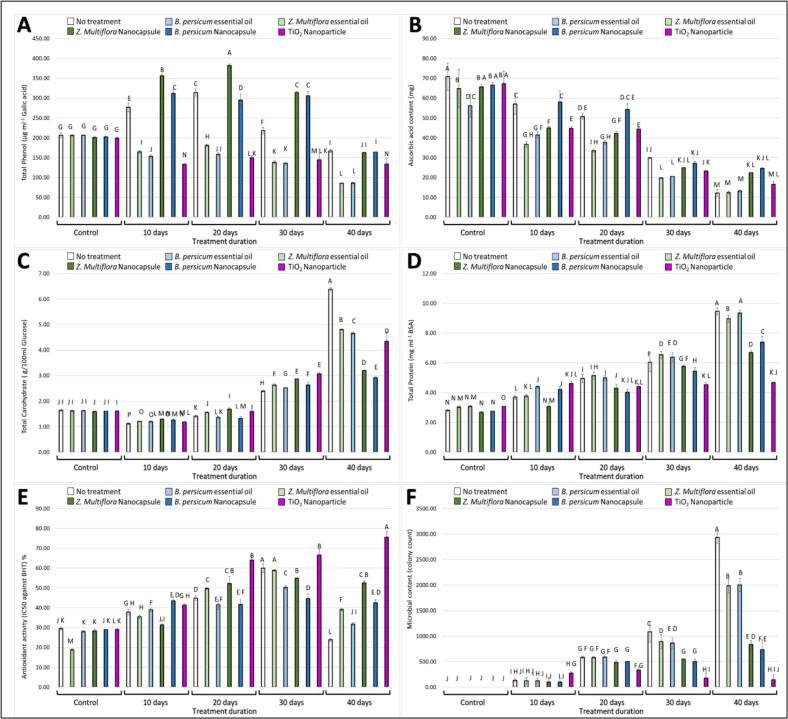
Effect of different treatments on various parameters of fresh-cut kale over different storage durations (0, 10, 20, 30, and 40 days). The figure includes the following measurements: **(A)** Total phenolic content, **(B)** Ascorbic acid content, **(C)** total carbohydrate content, **(D)** total protein content, **(E)** Antioxidant content, **(F)** microbial content. Treatments include no treatment (control), essential oils (*T*. *daenensis* Celak and *B. persicum*), nanoencapsulated essential oils (EO-NC), and TiO_2_ nanoparticle. Different letters represent significant differences between treatments.

Total phenolic content of fresh-cut kale is shown in Fig. 5A. Total phenolic content decreased with storage in all the groups (Fig. 5A). On day 20, phenolics in control samples decreased to approximately 3.0 mg GAE/g, whereas EO-NC and TiO₂ treatments retained 4.2 and 4.0 mg GAE/g, respectively. On day 40, the controls decreased further to ∼2.0 mg GAE/g, whereas EO-NC and TiO₂ treatments still retained greater amounts (3.4 and 3.2 mg GAE/g, respectively). Results indicate that EO-NC and TiO₂ usage slowed down decomposition of phenolic compounds and maintained far greater rates than controls (*T*. *daenensis* Celak and *B. persicum*). The EO-NC group presented the best maintenance in general, echoing a preventive effect against the oxidative degradation of phenolic compounds. This guarantees the success of encapsulation and nanoparticle treatments in the preservation of bioactive metabolites.

#### 3.4.2 Ascorbic acid (vitamin C)

Ascorbic acid content variation is depicted in Fig. 5B. As expected, the ascorbic acid content reduced over time in all treatment groups but with the least degradation in EO-NC and TiO₂-treated samples. The two treatment groups lost significantly less vitamin C content upon storage compared to control. Reduced ascorbic acid loss in treated samples can be explained by reduced exposure to oxidative stress and microbial breakdown, once again indicating the antioxidant-sparing activity of EO-NC and TiO₂ treatments.

#### 3.4.3 Total carbohydrate preservation

Total carbohydrate value is illustrated in Fig. 5C. Carbohydrate content declined progressively during storage (Fig. 5C). At day 20, carbohydrate content in controls reached ∼22 mg/g, whereas EO-NC and TiO₂ samples still had ∼32 and ∼ 30 mg/g, respectively. Controls declined to ∼15 mg/g by day 40, whereas EO-NC and TiO₂ still had more (27 and 25 mg/g). This confirms that EO-NC and TiO₂ treatments significantly reduce carbohydrate degradation compared with untreated kale. This verifies the delay of metabolism and cell wall breakdown in the treated samples that could be related to the conservational and antimicrobial effects of these chemicals. Of the treatments taken, EO-NC consistently revealed conservation of carbohydrate content.

#### 3.4.4 Total protein

Protein content values are shown in Fig. 5D. Protein content decreased in storage time in all groups (Fig. 5D). On day 20, control samples fell to ∼12 mg/g, while EO-NC and TiO₂ retained ∼18 and ∼ 17 mg/g, respectively. On day 40, controls fell to ∼9 mg/g, while EO-NC and TiO₂ recorded much higher values (15 and 14 mg/g). These results confirm again that nano-treatments retarded protein loss and enhanced preservation of nutritional quality. Preservation of protein integrity can be accounted for by reduced enzymatic breakdown, increased cellular stability, and antimicrobial safeguarding by these treatments. Interestingly, EO-NC treatment gave the highest protein retention on day 40.

#### 3.4.5 Antioxidant activity

Antioxidant activity of kale during storage is shown in Fig. 5E. As with other bioactive compounds, antioxidant capacity went down over time, but to the lowest degree in the EO-NC and TiO₂ treatment groups. The higher antioxidant retention in the two treatments could be explained by the additive effect of preserved phenolic compounds and ascorbic acid. Of all the interventions, EO-NC exerted the strongest impact on maintaining antioxidant activity, thereby validating its role in maintaining kale's functional quality at a high level during long-term storage.

#### 3.4.6 Microbial load and shelf-life extension

Microbial content profiles are shown in Fig. 5F. Control samples experienced exponential microbial load increase over time and spoiled sooner. In contrast, all treatments suppressed microbial growth to varying extents, with EO-NC and TiO₂ inhibiting the most microbially. Both treatments suppressed microbial growth significantly, thereby enhancing the shelf life of fresh-cut kale. In contrast, both EO-NC and TiO₂ treatments significantly suppressed microbial growth, with counts X log CFU/g lower than the control by day 40.

### 3.5 Antioxidant and antibacterial study of kale

Generally, findings in Fig. 5 (A–F) indicate that EO-NC and TiO₂ nanoparticles are extremely effective in sustaining the antioxidant character (total phenolics, ascorbic acid, antioxidant activity) and microbial contamination control of fresh-cut kale. Both of these treatments surpassed both the control and non-encapsulated essential oil treatment, indicating the added value through nanotechnology for extending the shelf life and nutritional content of plant food. The dual antioxidant and antibacterial activity of these treatments is a strategy for postharvest management of minimally processed vegetables.

### 3.6 Enzymatic activity

#### 3.6.1 Polyphenol oxidase

**Fig. 6:**
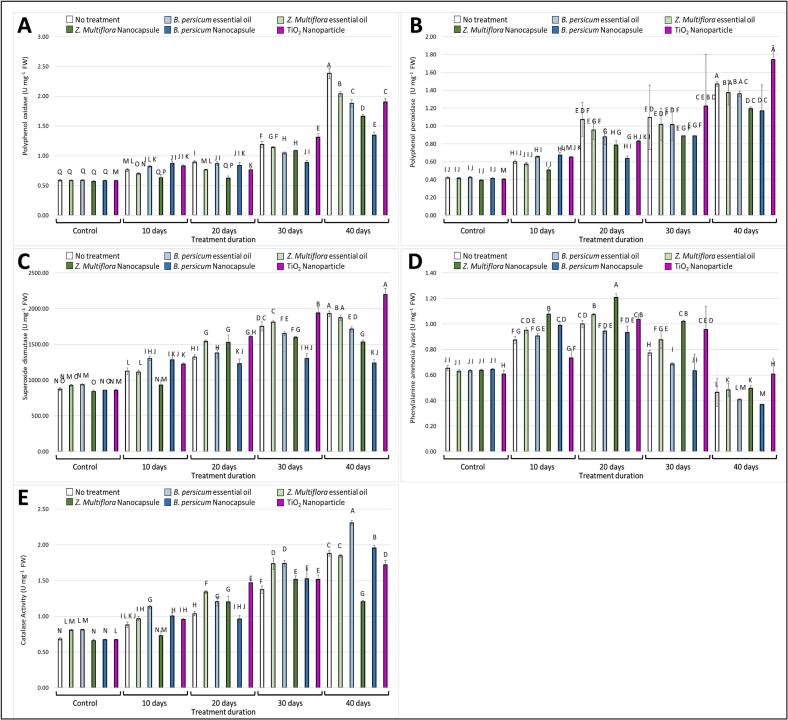
Effect of different treatments on various parameters of fresh-cut kale over different storage durations (0, 10, 20, 30, and 40 days). The figure includes the following measurements: **(A)** polyphenol oxidase activity, **(B)** polyphenol peroxidase activity, **(C)** superoxidase activity, **(D)** phenylalanine ammonia lyase activity, **(E)** catalase activity. Treatments include no treatment (control), essential oils (*T*. *daenensis* Celak and *B. persicum*), nanoencapsulated essential oils (EO-NC), and TiO_2_ nanoparticle. Different letters represent significant differences between treatments.

Polyphenol oxidase (PPO) activity increased sharply during storage in the control samples, reflecting enhanced enzymatic browning and tissue degradation (Fig. 6A). In contrast, both EO-NC and TiO₂ nanoparticle treatments significantly suppressed PPO activity, maintaining much lower levels throughout storage. Among all treatments, EO-NC exhibited the strongest inhibition of PPO, resulting in reduced browning and better preservation of visual and nutritional quality. Similarly, peroxidase (POD) activity increased progressively in control samples but remained relatively stable under EO-NC treatment, indicating the most consistent inhibition among treatments (Fig. 6B). This dual effect highlights the superior ability of EO-NCs—particularly due to their controlled release of bioactive compounds—to attenuate oxidative enzyme activities, either through direct enzyme inhibition or by reducing oxidative and microbial stress, thereby extending postharvest quality.

#### 3.6.2 Polyphenol peroxidase

The activity of polyphenol peroxidase (POD) is presented in Fig. 6B. POD activity in controls doubled by day 20 and peaked by day 40, while EO-NC treatments maintained levels close to baseline through day 30. This accounts for the fact that these treatments inhibited POD-catalyzed oxidative reactions, causing further degradation of phenolic compounds. EO-NC treatment showed the most stable inhibition, supporting its role in the regulation of enzymatic browning.

#### 3.6.3 Superoxide dismutase

Superoxide dismutase (SOD) activity is shown in Fig. 6C (designated as “superoxidase activity” in the figure legend). SOD activity, an indicator of antioxidant defense, initially increased and then progressively declined in all groups. Notably, EO-NC and TiO₂-treated samples exhibited greater SOD activity than the control at the later storage times. Greater SOD activity reflects higher stress response potential and higher protection against reactive oxygen species for the treated kale, resulting in longer freshness and biochemical stability.

#### 3.6.4 Phenylalanine ammonia-lyase

Results of phenylalanine ammonia-lyase (PAL) activity are shown in Fig. 6D. PAL is the key enzyme in the phenylpropanoid pathway of phenolic biosynthesis. In treated samples—especially those with EO-NC—PAL activity was significantly upregulated at the early and mid-storage periods compared to the control. Such elevated activity may be responsible for the higher phenolic content retained in those treatments, as stated in the above sections. The activation of PAL shows that EO-NC can induce plant defense mechanisms that result in enhanced shelf life and functional properties.

#### 3.6.5 Catalase

CAT activity is unveiled in Fig. 6E. CAT activity became successively reduced in control samples, reflecting the decline of antioxidant defense over time. But for EO-NC and TiO₂-treated kale, CAT activity was significantly higher over 40 days. This showed that these treatments helped in the preservation of enzymatic detoxification of hydrogen peroxide and also the protection of plant tissues against oxidative damage. EO-NC treatment, indeed, showed the highest retention of catalase activity, once again illustrating its antioxidant-enhancing ability.

## 4 Discussions

TiO₂ nanoparticles demonstrated strong potential as a postharvest treatment for maintaining the quality and extending the shelf life of fresh-cut kale. Compared with both the control and EO-NCs, TiO₂ treatments significantly reduced weight loss, improved water retention, and minimized color degradation during storage. While EO-NCs exhibited slightly superior chlorophyll retention, TiO₂ nanoparticles performed equally well or better across most other evaluated parameters, including the preservation of chlorophyll *a* and b and the maintenance of membrane integrity through reduced electrolyte leakage. These findings highlight TiO₂ nanoparticles as a reliable and long-lasting strategy for ensuring postharvest quality in fresh-cut kale. This research describes that EO-NC and TiO₂ packaging significantly enhanced the shelf life of fresh-cut kale under storage at 4 °C. Control samples exhibited widespread microbial spoilage and unacceptable appearance after ∼10–15 days, whereas EO-NC and TiO₂-treated samples registered negligible microbial growth, acceptable biochemical quality, and fresh appearance up to 30–40 days. Based on microbial load, sensory features, and nutrient retention (phenolics, vitamin C, and protein), the shelf life of fresh-cut kale was thus increased from ∼10–15 days in control untreated samples to ∼30–40 days with EO-NC or TiO₂ active packaging.

TiO₂ nanoparticle treatments demonstrated strong antioxidative and antimicrobial effects that contributed to improved postharvest quality of fresh-cut kale. Statistical analysis confirmed that TiO₂-treated samples retained higher levels of ascorbic acid, phenolics, and overall antioxidant activity compared with the control, reflecting enhanced oxidative stability. TiO₂ also exerted potent inhibitory effects on PPO, POD, and SOD activities, thereby reducing enzymatic browning and limiting oxidative damage. In addition, TiO₂ coatings significantly suppressed microbial proliferation, further supporting their role in spoilage prevention. Although EO-NCs provided slightly better chlorophyll retention, TiO₂ treatments performed equally well or better in most other parameters, including enzyme activity, mineral retention, and metabolite preservation. ANOVA analysis confirmed the highly significant (*p* < 0.01) effects of TiO₂ across multiple quality traits. Collectively, these findings highlight TiO₂ nanoparticles as a stable, safe, and efficient strategy for extending shelf life, minimizing quality losses, and maintaining the nutritional and visual attributes of leafy greens during storage.

**Fig. 7:**
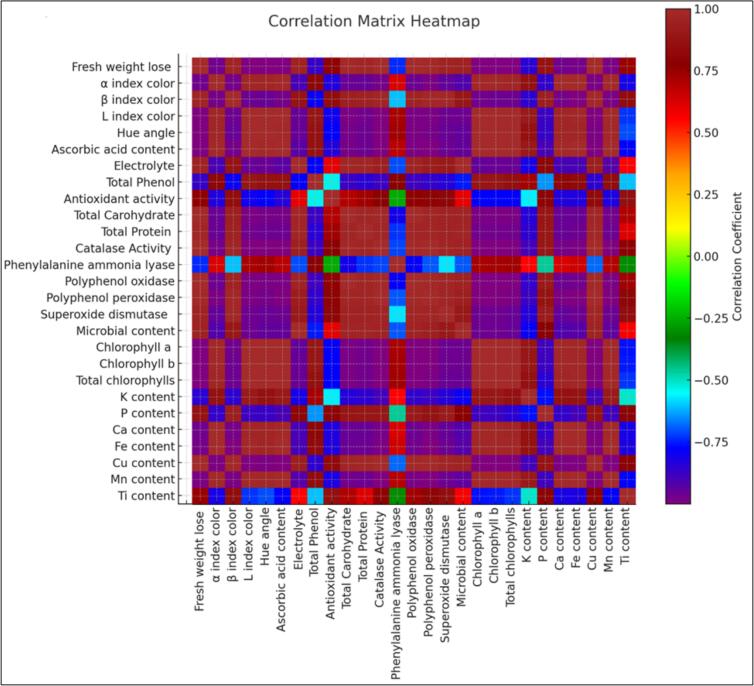
Heatmap analysis showing the changes in various parameters such as weight loss, chlorophyll content, ascorbic acid, antioxidant activity, etc. over time (0, 10, 20, 30, and 40 days). The data highlights the differences between various treatments (TiO_2_, EO-NC, control) and their effects on the preservation of kale quality throughout the storage period.

The heatmap analysis (Fig. 7) clearly indicates changes in parameters such as weight loss, chlorophyll content, ascorbic acid, and antioxidant activity among treatments. Of these, the TiO₂ and EO-NC treatments both had high preservation capacities, with the TiO₂ performing better in most quality factors.

The current research also corroborates earlier research on visual quality preservation of fresh-cut leafy greens with specific emphasis on kale. While nanocapsules of essential oils (EO-NCs) were found to be somewhat effective in visual attribute preservation, they were not as effective as titanium dioxide (TiO₂) nanoparticles. Although oregano and thyme essential oils were found to stabilize colors, their activity was much less compared to newer chemicals such as TiO₂, whose activity gave more stable and longer protection. Kale tissues treated with TiO₂ were found to retain more intense colors as indicated by a more stable difference value for brightness (ΔL) and reduced hue angle change, which indicates lesser color change(Naknaen, 2014; Zehra et al., 2022).

The TiO₂ treatment was significant in weight retention, chlorophyll stabilization, and nutrient preservation during storage. On day 40, TiO₂-treated samples had significantly lower weight loss compared to the control and EO-NC-treated samples, which suggests its use in reducing water loss by respiration and senescence. This is in conformity with new data on the barrier and photoprotective characteristics of TiO₂ preventing water loss and oxidative stress (de Fonseca et al., 2021). Color stability was improved by TiO₂ relative to EO-NC treatments as indicated by a more stable ΔL index and hue angle change with time. TiO₂ nanoparticles are defined by UV blocking, and this type of effect is able to retard pigment breakdown in green vegetables, in line with a finding quoted by Herrera-Rivera et al., whereby there was improved visual quality and senescence delay of TiO₂ coating-treated vegetables. Retention of ascorbic acid was also preserved in TiO₂-treated kale, the retention levels being far from control and slightly better than EO-NC samples. This is because TiO₂ is an antioxidant as well as a photocatalyst, which suppresses oxidative degradation by quenching ROS (del Herrera-Rivera et al., 2024).s

In nutrient retention, TiO₂ nanoparticles again prevailed over EO-NC treatments. Ascorbic acid, a valuable antioxidant found in kale, was retained much more in TiO₂-treated samples. This suggests that the nanoparticles form a robust barrier, limiting oxygen penetration and helping to preserve vitamin C content. EO-NCs, particularly those with *T*. *daenensis* Celak and *B. persicum*, also supported antioxidant stability but to a lesser degree. In addition to ascorbic acid, TiO₂ treatments also led to improved retention of phenolic compounds and antioxidant activity. This is suggestive of the multifunctionality of TiO₂ nanoparticles, not only protecting against oxidative stress but also maintaining the nutritional value of fresh produce. The EO-NCs partially succeeded in maintaining such bioactive compounds but were less consistent in maintaining levels of antioxidants during the storage period. Chlorophyll content, which is a critical predictor of freshness and market value of leafy greens, was likewise best maintained in the TiO₂-treated batch. Their samples manifested intense green color and slowed down chlorophyll degradation. EO-NC-treated samples likewise retained some chlorophyll but to a far lesser degree than that of the TiO₂ batch. Control samples, on the other hand, exhibited quick color loss and senescence symptoms, implying absence of oxidative protection. These findings are also supported by Li and Machanuru et al. (Machanuru et al., 2024), who demonstrated that TiO₂ coatings are effective in inhibiting ROS accumulation, resulting in prolonged stability of ascorbic acid in treated vegetables.

The heatmap in Fig. 7 emphasizes the evidence of TiO₂ treatments in maintaining antioxidant activity over extended periods. This can be explained through the capacity of TiO₂ to maintain bioactive molecules in storage conditions. Besides that, enzymatic browning spoilage and activity, i.e., catalase (CAT) and polyphenol oxidase (PPO) were most limited in the TiO₂ group. These denote a lower rate of browning due to enzymatic activity as well as decreased degradation, conducive to a longer shelf life. There were moderately decreased enzymatic activities present in the EO-NC-treated samples but weaker in action. Enzymatic activity was maximum in the control group, which goes hand-in-hand with higher rates of spoilage. Microbial load, a crucial food safety criterion, was also minimum in TiO₂-treated samples. This is proof for the superb antibacterial activity of TiO₂, which has been attributed to its capability in destabilizing microbial membranes and inhibiting bacterial growth. While EO-NCs, especially those that include *T*. *daenensis* Celak, also exhibited antimicrobial activity, their ability to resist microbial growth was not as effective as in the case of TiO₂ nanoparticles. While treatments using EO-NCs had sustained antioxidant capacity, particularly with *T*. *daenensis* Celak EO, to a lesser extent, their activity was not quite as remarkable overall. This is also in agreement with current literature on essential oils being stated to be antioxidant in potency but without long-term protection as well as barrier function (Salehi et al., 2012). Chlorophyll content, critical to consumer acceptability and freshness, was stabilized most in the TiO₂ treatment group (12.61 mg/g), followed by EO-NC treatments, and least in the control group (8.25 mg/g). The retention of chlorophyll in the TiO₂-treated samples indicates that the treatment was effective in preventing the degradation and senescence of chlorophyll, which is consistent with current literature that confirmed that TiO₂ nanoparticles shield leaf tissue against oxidative and photodegradative stress (Wang et al., 2023). Enzyme activity, particularly of catalase (CAT), polyphenol oxidase (PPO), and peroxidases, was minimum for the TiO₂-treated samples, demonstrating effective inhibition of oxidative metabolism. The control treatment had maximum enzyme activity, confirming uncontrolled oxidative stress and increased spoilage. Intermediate activity levels were observed for EO-NC treatments. These results confirm TiO₂’s effectiveness in inhibiting enzymatic browning and deterioration, as reported in a study by Xing et al. (Xing et al., 2021), where TiO₂ coatings inhibited browning enzymes in vegetables stored for longer periods. Microbial growth was also limited in TiO₂-treated samples (130 CFU/mL), indicating strong antimicrobial activity. This antimicrobial activity could be ascribed to the property of TiO₂ to generate ROS upon exposure to light, which disintegrates microbial cell walls and inhibits proliferation. These findings are in agreement with Xing et al. (Xing et al., 2021), who documented strong antimicrobial activities of TiO₂-based films in postharvest application.

This study demonstrates that both TiO₂ nanoparticles and EO-NCs effectively enhanced the postharvest quality of fresh-cut kale, though their mechanisms and relative strengths differed. TiO₂ nanoparticles provided broad-spectrum and long-lasting preservation through their multifunctional antioxidative, antimicrobial, and barrier properties, resulting in significant improvements in weight retention, chlorophyll stabilization, nutrient preservation, and suppression of enzymatic browning and microbial growth. EO-NCs, particularly those incorporating *T*. *daenensis* Celak, were especially effective in maintaining membrane integrity and controlling oxidative enzyme activity, offering targeted antioxidant and antimicrobial protection. Importantly, the combination of a photocatalytic nanoparticle with sustained-release antioxidant nanocapsules represents a promising synergistic strategy for postharvest applications, in which the structural stability and antimicrobial activity of TiO₂ complement the controlled antioxidant release of EO-NCs. Together, these findings highlight the potential of TiO₂- and EO-NC-based active packaging systems as innovative, safe, and multifunctional approaches to extend shelf life, maintain nutritional quality, and preserve consumer acceptability of fresh-cut leafy greens.

## 5 Conclusions

This study confirmed that both titanium dioxide (TiO₂) nanoparticles and nanoencapsulated essential oils (EO-NCs) are effective postharvest treatments for maintaining the quality of fresh-cut kale during prolonged storage at 4 °C. Compared with untreated controls, both treatments significantly reduced weight loss, delayed chlorophyll degradation, preserved essential nutrients (ascorbic acid, phenolics, minerals), and suppressed microbial growth. TiO₂ nanoparticles consistently outperformed EO-NCs across most parameters, particularly in reducing oxidative stress, limiting enzymatic browning, and enhancing antioxidant stability. EO-NCs, especially those containing *T*. *daenensis* Celak essential oil, showed particular strength in maintaining membrane integrity and inhibiting browning-related enzyme activities. Encapsulation clearly improved the stability and efficacy of essential oils compared with their free forms. Overall, TiO₂ and EO-NC treatments represent promising approaches for extending shelf life and preserving the nutritional and sensory quality of fresh-cut kale. Future studies should explore their combined application to exploit potential synergistic effects, while also addressing critical issues such as consumer sensory acceptance, migration behavior, toxicological safety, and the relatively high cost of nano-enabled packaging materials. Addressing these limitations is essential to enable the safe, sustainable, and scalable use of TiO₂- and EO-NC-based active packaging technologies in the food industry.

## CRediT authorship contribution statement

**Faezeh Mirzajani:** Writing – original draft, Methodology, Data curation, Conceptualization. **Raheleh Ebrahimi:** Writing – review & editing, Validation, Supervision. **Weria Weisany:** Writing – review & editing, Validation, Software, Project administration, Methodology, Investigation, Funding acquisition, Formal analysis, Data curation. **Orang Khademi:** Writing – review & editing.

## Declaration of competing interest

The authors declare that they have no known competing financial interests or personal relationships that could have appeared to influence the work reported in this paper.

## Data Availability

Data will be made available on request.
